# Effects of Arsenite Exposure during Fetal Development on Energy Metabolism and Susceptibility to Diet-Induced Fatty Liver Disease in Male Mice

**DOI:** 10.1289/ehp.1409501

**Published:** 2015-07-07

**Authors:** Eric J. Ditzel, Thu Nguyen, Patricia Parker, Todd D. Camenisch

**Affiliations:** 1 Department of Pharmacology and Toxicology, College of Pharmacy,; 2 Steele Children’s Research Center,; 3 Southwest Environmental Health Sciences Center,; 4 Sarver Heart Center, and; 5 Bio5 Institute, University of Arizona, Tucson, Arizona, USA

## Abstract

**Background:**

Chronic exposure to arsenicals at various life stages and across a range of exposures has been implicated in cardiometabolic and liver disease, but disease predisposition from developmental exposures remains unclear.

**Objectives:**

*In utero* and post-weaning exposure to trivalent arsenic (As^III^) was examined on the background of a Western-style diet to determine whether As^III^ exposure affects metabolic disease.

**Methods:**

Male Swiss Webster mice were exposed to 100 ppb As^III^ *in utero*, after weaning, or both. *Ad libitum* access to a Western-style diet was provided after weaning, and the plasma metabolome, liver histopathology, liver enzyme activity, and gene expression were analyzed.

**Results:**

Hepatic lipid composition and histopathology revealed that developmental As^III^ exposure exacerbated Western-style diet–induced fatty liver disease. Continuous As^III^ exposure increased cardiometabolic risk factors including increased body weight, insulin resistance, hyperglycemia, and plasma triglycerides. As^III^ exposure produced a decrease in the intermediates of glycolysis and the TCA cycle while increasing ketones. Hepatic isocitrate dehydrogenase activity was also decreased, which confirmed disruption of the TCA cycle. Developmental As^III^ exposure increased the expression of genes involved in fatty acid synthesis, lipogenesis, inflammation, and packaging of triglycerides, suggesting an increased acetyl coenzyme A (acetyl-CoA) load.

**Conclusions:**

*In utero* and continuous early-life exposure to As^III^ disrupted normal metabolism and elevated the risk for fatty liver disease in mice maintained on a high-fat diet. Our findings suggest that individuals exposed to As^III^ during key developmental periods and who remain exposed to As^III^ on the background of a Western-style diet may be at increased risk for metabolic disease later in life.

**Citation:**

Ditzel EJ, Nguyen T, Parker P, Camenisch TD. 2016. Effects of arsenite exposure during fetal development on energy metabolism and susceptibility to diet-induced fatty liver disease in male mice. Environ Health Perspect 124:201–209; http://dx.doi.org/10.1289/ehp.1409501

## Introduction

Chronic arsenic exposure has become increasingly prevalent with a shift from the use of surface water to the drilling of wells to reach “cleaner” water. The use of well water increases the risk for individuals to be exposed to arsenic (Yoshida et al. 2004). Exposure to arsenicals increases mortality from cardiovascular disease and hypertension in populations exposed to these compounds during gestation as well as into adulthood (Hawkesworth et al. 2013; Yuan et al. 2007). Cardiometabolic syndrome is a set of metabolic dysfunctions combined with increased blood pressure that culminates in an increased risk for cardiovascular disease (Kirk and Klein 2009). The relationship between metabolic syndrome and arsenic has been strengthened over the years, but conflicting results have appeared in recent epidemiological studies (Bräuner et al. 2014; Chen et al. 2010). Factors contributing to this discrepancy could include variables such as regional differences in nutrition. In addition, only a few studies have focused on the impact of *in utero* and early-life exposures to low-level arsenicals, and the need to investigate this type of exposure has been highlighted by the National Institute of Environmental Health Sciences (NIEHS) (Dávila-Esqueda et al. 2011; Maull et al. 2012; States et al. 2012).

The majority of studies investigating nonalcoholic fatty liver disease (NAFLD), from benign steatosis to end-stage liver disease in nonalcoholic steatohepatitis (NASH), have principally focused on arsenical exposures in parts per million ranges. Although environmentally relevant in some areas, investigations considering only these levels of exposure have left much uncertainty about the potential effects of chronic exposures in the parts per billion range (Arteel et al. 2008; Reilly et al. 2014; Shi et al. 2014; Tan et al. 2011). In addition, our laboratory has demonstrated incidences of NAFLD in mice with low-level *in utero* exposure to arsenicals, and the present work aims to expand on those initial findings (Sanchez-Soria et al. 2014). The incidence of NAFLD is important to consider when examining cardiometabolic disease because NAFLD is thought to be the hepatic manifestation of metabolic syndrome (Paschos and Paletas 2009). There is also an association with elevated mediators of atherosclerosis in patients with NALFD that suggests a link to cardiovascular disease (Sookoian et al. 2010).

The objective of this study was to examine the effects of exposure to low levels (100 ppb) of trivalent arsenic (As^III^) on mice that were exposed during gestation, after weaning, or throughout life, with all mice being exposed to a Western-style diet after weaning. We have previously shown that low-level exposure to As^III^] *in utero* was associated with incidence of fatty liver disease; this study aimed to reproduce these results on the background of a high-fat diet to determine whether As^III^ exposure contributes to the incidence and severity of NAFLD (Sanchez-Soria et al. 2014). In addition, components of cardiometabolic syndrome were investigated to examine how these disease risk factors may be affected by low-level As^III^ exposure.

## Materials and Methods

*Animals and treatment.* Primi-pregnant Carworth Farms Swiss Webster mice were purchased from Charles River Laboratories and housed individually in sterile microisolator cages (12.7 cm high with 483.87 cm^2^ of floor) with corncob bedding (7097.25 Corncob; Harlan Laboratories Inc.) on a 12-hr light/dark cycle at 20°C. This mouse model was used because it has no known metabolic or cardiovascular disease predisposition that could have affected results. Food (2019 Teklad Global 19% Protein Extruded Rodent Diet; Harlan Laboratories Inc.) and water were provided *ad libitum* with either 100 ppb arsenite [as sodium arsenite (NaAsO_2_), Sigma] or 100 ppb sodium chloride (NaCl, VWR). The Arizona Laboratory for Emerging Contaminants verified the arsenite concentrations by inductively coupled plasma mass spectrometry. Control mice received 100 ppb NaCl in water; to induce *in utero* (IU) exposure, dams (53 days old) received 100 ppb NaAsO_2_ in water beginning at 5 days postfertilization [embryonic day 5 (ED5)] through birth; dams of the *in utero* and continuous (IU+) mice received 100 ppb NaAsO_2_ in water beginning at ED5, and pups continued on 100 ppb NaAsO_2_ in water postnatally until the end of the study at 13 weeks, when all animals were sacrificed via carbon dioxide (CO_2_) euthanasia followed by cervical dislocation. Postnatal (PN) mice received 100 ppb NaAsO_2_ in water starting after weaning through the end of the study.

Out of nine total litters, six were exposed to As^III^ *in utero* and three were exposed to 100 ppb NaCl. The PN and CTRL males were drawn from the three untreated litters, and the six remaining *in utero* As^III^ exposed litters were broken into two groups of three litters (IU and IU+). Litter information is included in Supplemental Material, Table S1. For the exposure groups, CTRL *n* = 10, IU *n* = 14, IU+ *n* = 13, and PN *n* = 5. Litter contributions to exposure groups are included in Supplemental Material, Table S2. Water was replaced weekly. Before weaning, pups remained with their own mothers in one cage regardless of litter size. After weaning (21 days old), mice were provided with Western Diet (TestDiet) *ad libitum* and housed with the same exposure group four per cage; efforts were made to house littermates together. This report focuses solely on the male cohort.

The Western-style diet utilized in this study (15.5% protein, 40.1% fat, 44.4% carbohydrate by kilocalorie, with the primary ingredients being 34.05% sucrose, 19.97% milk fat, and 19.47% casein) is similar to the standard American diet (15% protein, 33% fat, 50% carbohydrate by kilocalorie) (Grotto and Zied 2010). Body weights were measured weekly in the morning, and blood plasma was collected at weaning, 5 weeks, and 9 weeks via retro-orbital (RO) bleeding after a 6-hr fast starting at 0600 hours, with bleeds carried out at 1200 hours. All animal use and experimental protocols followed University of Arizona Institutional Animal Care and Use Committee (IACUC) regulations and remained in accordance with institutional guidelines, ensuring that animals were treated humanely and with regard for alleviation of suffering.

*Metabolomic analysis.* Plasma from week 9 RO bleeds (minimum of seven samples per group) was subjected to metabolomic analysis (Metabolon®). A total of 337 compounds of known identity were queried with a combination of liquid chromatography–mass spectrometry (LC-MS), liquid chromatography–tandem mass spectrometry (LC-MS/MS), and gas chromatography–mass spectrometry (GC-MS) techniques and were examined for significant alterations as described previously (Ganti et al. 2012).

*Histology and pathology.* Animal tissues were isolated and rinsed in cold 1× phosphate-buffered saline, and total liver weight was measured. The caudate liver lobe was embedded in Tissue-Tek O.C.T.™ compound (Sakura) and frozen in liquid nitrogen vapor. The median lobe was fixed in 100% ethanol, 37% formaldehyde, and 100% glacial acetic acid in a 6:3:1 (volume/volume/volume) ratio for 4 hr at RT and then overnight at 4°C with fresh fixative; both steps were performed with slow agitation. Frozen livers were sectioned to 10 μm on a cryostat and fixed with 10% buffered formalin. The sections were stained using the Oil Red O in propylene glycol method for lipid detection (Poly Scientific R&D Corp.). Fixed livers were processed in Paraplast 56 (McCormick Scientific), and sectioned to 10 μm. Sections were stained with hematoxylin and eosin (H&E) (Thermo Scientific) or Picrosirius Red: 0.1% Direct Red 80 (Sigma) and a saturated aqueous solution of picric acid. Oil Red O and H&E sections were imaged using a bright-field microscope, and Picrosirius Red sections were imaged using a crossed-polarization light microscope. H&E sections were scored under blinded conditions utilizing the NAFLD activity score (NAS): The score is defined as the unweighted sum of the scores for steatosis (0–3), lobular inflammation (0–3), and ballooning (0–2); scores of 0–2 are not NASH, 3–4 are considered borderline, and 5–8 are considered NASH (Kleiner et al. 2005).

*Reverse transcription real-time polymerase chain reaction (RT-qPCR).* RNA was isolated and purified from flash-frozen tissue samples using TRIzol® according to the manufacturer’s protocol (Life Technologies). First-strand cDNA was generated using a Transcriptor First Strand cDNA Synthesis kit (Roche) with 1 μg RNA. Real-time polymerase chain reaction (qPCR) was performed using the TaqMan Master Primer-Probe System (Roche), and 40S ribosomal protein 7 (*Rps7*) was used as a housekeeping gene for relative quantification of the target mRNA. Table 1 lists the genes of interest, the primer sequences, and the corresponding proprietary fluorescein-labeled probes from Roche’s Universal ProbeLibrary.

**Table 1:** Primers and probes for RT-qPCR.

Gene	Left (5’-3’)	Right (5’-3’)	Probe	NCBI RefSeq
*Acaca*	GGCTCAAACTGCAGGTATCC	TTGCCAATCCACTCGAAGA	103	NM_133360.2
*Apob*	GAGAACTTCGCTGCTTCCAA	CAGCAGTGCACTTTGCGTAG	42	NM_009693.2
*Cd36*	TTGTACCTATACTGTGGCTAAATGAGA	CTTGTGTTTTGAACATTTCTGCTT	9	L23108.1
*Cpt1a*	GACTCCGCTCGCTCATTC	TCTGCCATCTTGAGTGGTGA	70	NM_013495.2
*Dgat1*	TCGTGGTATCCTGAATTGGTG	AGGTTCTCTAAAAATAACCTTGCATT	9	NM_010046.2
*Il6*	GCTACCAAACTGGATATAATCAGGA	CCAGGTAGCTATGGTACTCCAGAA	6	NM_031168.1
*Mttp*	GCCCAACGTACTTCTAATTTATGG	TGCTGGCCAACACGTCTA	55	NM_001163457.1
*Pparg*	TGCTGTTATGGGTGAAACTCTG	CTGTGTCAACCATGGTAATTTCTT	2	NM_011146.3
*Ppargc1a*	GAAAGGGCCAAACAGAGAGA	GTAAATCACACGGCGCTCTT	29	NM_008904.2
*Rps7*	AGCACGTGGTCTTCATTGCT	CTGTCAGGGTACGGCTTCTG	101	NM_011300.3
*Slc27a2*	GCGTGCCTCAACTACAACATT	CCTCCTCCACAGCTTCTTGT	84	NM_011978.2
*Srebf1*	GGTTTTGAACGACATCGAAGA	CGGGAAGTCACTGTCTTGGT	78	NM_011480.3
*Tgfb1*	TGGAGCAACATGTGGAACTC	GTCAGCAGCCGGTTACCA	72	NM_011577.1
*Tnf*	CTGTAGCCCACGTCGTAGC	TTGAGATCCATGCCGTTG	25	NM_013693.2
The examined genes are acetyl-CoA carboxylase 1 (*Acaca*), apolipoprotein B-100 (*Apob*), platelet glycoprotein 4 (*Cd36*), carnitine O-palmitoyltransferase 1, liver isoform (*Cpt1A*), diacylglycerol O-acyltransferase 1 (*Dgat1*), interleukin-6 (*Il6*), microsomal triglyceride transfer protein large subunit (*Mttp*), peroxisome proliferator-activated receptor gamma (*Pparg*), peroxisome proliferator-activated receptor gamma coactivator 1-alpha (*Ppargc1a*), 40S ribosomal protein S7 (*Rps7*), very long-chain acyl-CoA synthetase (*Slc27a2*), sterol regulatory element-binding protein 1 (*Srebf1*), transforming growth factor beta-1 (*Tgfb1*), and tumor necrosis factor (*Tnf*).				

*Liver lipid content and enzymatic activity analysis.* Lipids from 25 mg of flash-frozen liver tissue were extracted with 500 μL chloroform:methanol in a 2:1 volume ratio. The lipid fraction was resuspended in 1% Triton X-100 in 100% ethanol. Triglycerides (TAGs) and cholesterol were quantified using a TAG reagent set and a cholesterol reagent set, respectively (GPO Reagent Set; Pointe Scientific). Free fatty acids (FFAs) were quantified using a colorimetric free fatty acid assay kit [Free Fatty Acid Assay Kit (Colorimetric); Cell Biolabs] according to the manufacturer’s protocol. Samples from flash-frozen liver tissue were analyzed using the following kits according to the manufacturers’ instructions: Aspartate Transaminase (AST) Activity Assay Kit and Isocitrate Dehydrogenase (IDH) Activity Assay Kit (Sigma) and Alanine Transaminase (ALT) Colorimetric Activity Assay Kit (Cayman Chemical).

*Blood biochemistry.* Plasma samples were analyzed with the following kits according to the manufacturers’ instructions: Free Fatty Acid Fluorometric Assay Kit and Glucose Colorimetric Assay Kit (Cayman Chemical), Rat/Mouse Insulin ELISA Kit (Millipore), and Mouse Hemoglobin A1c (HbA1c) Assay Kit (CrystalChem). Insulin resistance was determined using the homeostasis model assessment of insulin resistance (HOMA-IR) (Matthews et al. 1985).

*Statistical analysis.* For all experiments, aside from the metabolomic analysis (described above), a sample set with a minimum of five animals per treatment group in analytical duplicates was used, and a one-way analysis of variance (ANOVA) followed by Dunnett’s multiple comparison test was performed (Prism 6; GraphPad Software, Inc.) for comparisons between treatment groups and controls. The reported *p*-value is the multiplicity-adjusted *p*-value corrected for multiple comparisons.

## Results

**Figure 1:**
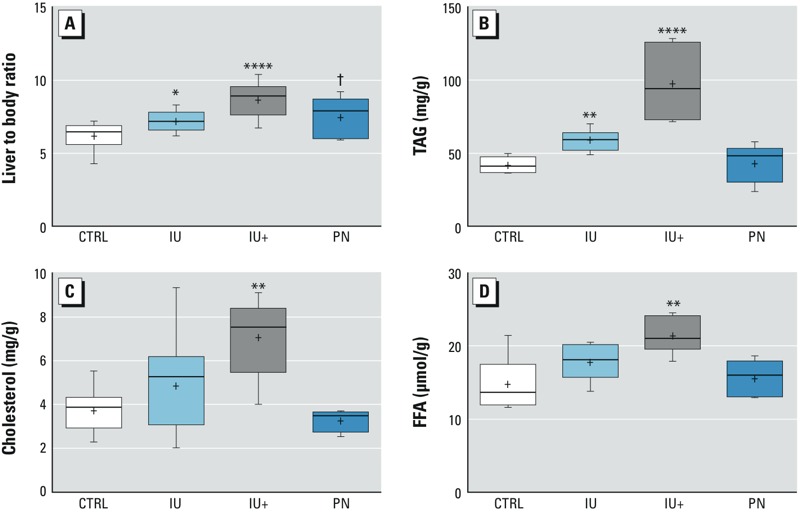
Effects of As^III^ exposure on liver weight and hepatic lipid content. (*A*) Increased proportional liver weight with As^III^ treatment at sacrifice. (*B*) Increased triglycerides in IU and IU+ livers. (*C*) Increased cholesterol and (*D*) increased free fatty acids in IU+ livers only. The box extends from the 25th to the 75th percentile, the whiskers indicate the entire distribution, the line is the median, and the + indicates the mean. (*A*) CTRL *n *= 10, IU *n *= 14, IU+ *n *= 13, PN *n *= 5; (*B,C*) CTRL *n *= 9, IU *n *= 14, IU+ *n *= 6, PN *n *= 5; (*D*) CTRL, IU, and IU+ *n *= 6, PN *n *= 5. ****p ≤ 0.0001, **p ≤ 0.01, *p ≤ 0.05, †*p* ≤ 0.1 compared with controls.

**Figure 2:**
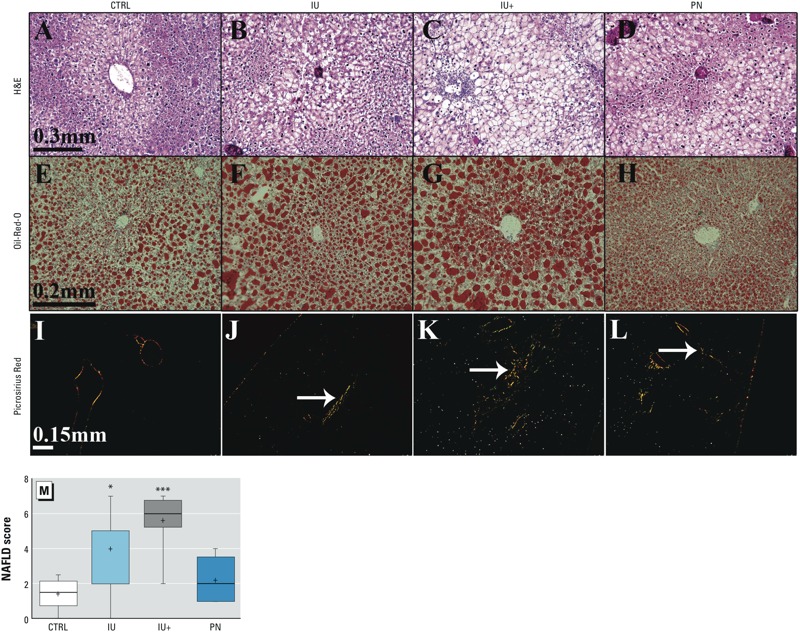
Effects of As^III^ on lipid accumulation, hepatocellular damage, and fibrosis. Rows represent each different stain utilized on sections prepared at sacrifice, and columns contain a representative section from each treatment group. The first row, H&E staining (0.3-mm bar for scale), shows various degrees and distribution of steatosis, inflammation, and hepatocellular ballooning. In the second row, Oil Red O (0.2-mm bar for scale), lipids are stained red, indicating increased lipid accumulation across the As^III^ treatment groups. In the third row, Picrosirius Red (0.15-mm bar for scale), white arrows highlight birefringence specific for collagen, indicating fibrosis. NAS scoring of the H&E sections (*M*) indicated little to no NASH in the CTRL and PN groups, equivocal NASH in the IU group, and definitive NASH in the IU+ group. The box extends from the 25th to the 75th percentile, the whiskers indicate the entire distribution, the line is the median, and the + indicates the mean. (*A–M*) CTRL *n *= 6, IU *n *= 7, IU+ *n *= 8, PN *n *= 5; ***p ≤ 0.001, *p ≤ 0.05 compared with controls.

*Effects of As^III^ on hepatic lipids and tissue damage. In utero* As^III^ exposure increased liver weight as a proportion of body weight at 13 weeks of age, and this increase in weight was accompanied by elevated TAGs in livers (Figure 1A,B). Only mice that were continuously exposed to As^III^ had increased hepatic FFAs and cholesterol (Figure 1C,D). Mice exposed to As^III^ had increased hepatocellular ballooning degeneration, increased lipid content, and fibrosis when compared to animals that were not exposed to arsenic. H&E staining of liver sections (Figure 2A–D) revealed severe NAFLD in mice exposed to As^III^ during embryonic development. Steatosis is considered more severe as it progresses outward from the centrilobular region to zone 1, and it was apparent in all mice, but lipid accumulation was more panacinar in *in utero* As^III^ exposed groups (Figure 2B,C), whereas it was more limited to zones 3 and 2 in control and PN animals (Figure 2A,D). In addition, more severe hepatocellular ballooning and inflammatory lesions were observed in the IU and IU+ groups than in the other groups. Oil Red O Staining was used to differentiate lipids in frozen liver sections, and the most severe lipid accumulation was detected in the IU and IU+ livers (Figure 2F,G), with mild to moderate lipid accumulation occurring in the control and PN livers (Figure 2E,H). Picrosirius Red staining of liver sections under polarized light revealed no incidences of fibrosis in the livers of control mice (Figure 2I); in contrast, a number of fibrotic lesions were detected in all As^III^-exposed groups (Figure 2J–L). Fibrotic lesions were generally focal and < 0.5 μm in diameter. Blinded scoring of the H&E sections (Figure 2M) showed that the NAFLD scores of the control and PN groups did not suggest NASH, that the IU group had scores that were borderline, and that the IU+ group had definitive NASH. The IU and IU+ groups had significantly higher NAFLD scores than the control animals. Taken together, these results indicate the presence of NAFLD in the groups that were exposed to As^III^ during development, with the most severe pathology developing with continuous exposure to As^III^ after birth.

**Figure 3:**
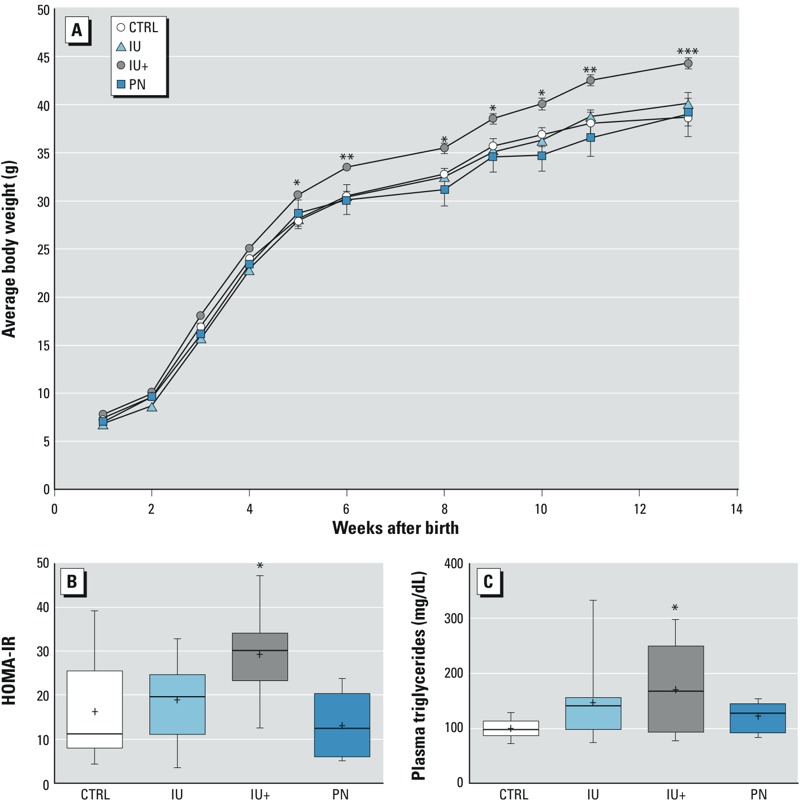
As^III^ exposure and cardiometabolic risk factors. The IU+ treatment group had (*A*) increased body weight 5 weeks after birth, (*B*) increased insulin resistance at week 5, and (*C*) increased circulating triglycerides at week 5. Error bars in *A* represent the standard error. The box extends from the 25th to 75th percentiles, the whiskers show the entire distribution, the line is the median, and the + indicates the mean. (*A*) At 13 weeks, CTRL *n *= 10, IU *n *= 14, IU+ *n *= 13, PN *n *= 5; (*B,C*) CTRL *n *= 10, IU and IU+ *n *= 11, PN *n *= 7; ***p ≤ 0.001, **p ≤ 0.01, *p ≤ 0.05 compared with controls.

*As^III^ exposure and cardiometabolic risk factors.* IU+ exposure to As^III^ resulted in an increase in cardiometabolic risk factors. IU+ mice gained more weight than controls at week 5 and remained heavier until the conclusion of the study at week 13. No statistically significant changes in weight were detected in any of the other As^III^ exposure groups (Figure 3A). Insulin resistance and plasma triglyceride levels were significantly higher in IU+ mice than in controls (Figure 3B,C). As^III^ exposure did not affect plasma FFAs at 5 weeks of age or blood HbA1c, a long-term marker of blood glucose, at 13 weeks of age (data not shown). Although As^III^ did not affect the HbA1c or FFA concentrations, continuous As^III^ exposure did increase body weight, insulin resistance, and serum TAGs, all of which are cardiometabolic risk factors.

*Effects of As^III^ on lipid and glucose metabolism.* The abovementioned data suggest that exposure to As^III^ during embryonic development primes animals for the development of cardiometabolic disease risk factors and NAFLD when exposure continues throughout the animal’s life. Therefore, As^III^-induced alterations in energy metabolism were investigated by metabolomic analysis of plasma samples from the mice to identify changes that may contribute to metabolic disease. Table 2 highlights biochemicals in plasma that were found to be substantially altered in As^III^-exposed mice but not in the controls. For example, 1,5-anhydroglucitol was decreased only in the IU+ mice but was not decreased in the controls. This depletion suggests recent (< 1 month) hyperglycemia (Dungan 2008). In addition, key intermediates in and end products of glycolysis were differentially altered depending on the exposure period. Intermediates within the TCA cycle were decreased primarily in plasma from mice exposed to As^III^ *in utero*, suggesting a potential disruption of oxidative energy metabolism. Increased ketone bodies were detected in the IU and IU+ mice, indicated by significant increases in both beta-hydroxybutyrate and acetoacetate. In addition, a few long-chain polyunsaturated fatty acids (LCPUFAs) were decreased in the IU group; LCPUFAs can be modulators of lipid metabolism. Taken together, these data suggest that As^III^ exposure has diverse and profound effects on energy metabolism, including alterations in glycolysis, the TCA cycle, and ketogenesis.

**Table 2:** Effects of As^III^ exposure on the energy metabolism profile.

Metabolite	IU	IU+	PN	CTRL	IU	IU+	PN
1,5-Anhydroglucitol	0.97	0.61*	0.99	1.08 ± 0.29	1.04 ± 0.34	0.66 ± 0.40	1.07 ± 0.34
3-Phosphoglycerate	0.62†	0.34*	0.4*	2.32 ± 1.14	1.44 ± 1.10	0.79 ± 0.27	0.92 ± 0.42
2,3-Diphosphoglycerate	0.51	0.29†	0.2*	3.96 ± 3.44	2.03 ± 2.43	1.16 ± 1.46	0.79 ± 0.64
Phosphoenolpyruvate (PEP)	0.42*	0.28*	0.17*	3.87 ± 3.06	1.62 ± 3.09	1.10 ± 1.50	0.65 ± 0.33
Pyruvate	0.81	1.19	1.64*	0.93 ± 0.40	0.76 ± 0.20	1.12 ± 0.54	1.54 ± 0.42
Lactate	0.67*	0.74*	1.01	1.30 ± 0.28	0.87 ± 0.12	0.96 ± 0.20	1.32 ± 0.47
Succinate	0.64†	0.86	1	1.15 ± 0.39	0.74 ± 0.14	0.99 ± 0.34	1.15 ± 0.45
Fumarate	0.71†	0.61*	0.61*	1.39 ± 0.42	0.99 ± 0.35	0.85 ± 0.30	0.84 ± 0.25
Malate	0.45*	0.55*	0.71	1.70 ± 0.66	0.77 ± 0.23	0.93 ± 0.53	1.21 ± 0.58
β-Hydroxybutyrate	5.07*	5.5*	2.06	0.42 ± 0.14	2.11 ± 1.85	2.29 ± 1.03	0.86 ± 0.95
Acetoacetate	4.81*	4.88*	1.56	0.56 ± 0.37	2.68 ± 1.67	2.72 ± 1.52	0.87 ± 0.79
Dihomo-linoleate (20:2n6)	0.65*	0.88	1.05	1.09 ± 0.32	0.71 ± 0.16	0.96 ± 0.27	1.14 ± 0.35
Mead acid (20:3n9)	0.67*	0.97	1.18	1.17 ± 0.37	0.78 ± 0.17	1.13 ± 0.42	1.38 ± 0.35
Docosadienoate (22:2n6)	0.65*	0.83	0.94	1.12 ± 0.31	0.73 ± 0.22	0.93 ± 0.25	1.05 ± 0.19
Plasma metabolomic analysis showed that select metabolites (with a focus on energy metabolism) were statistically significantly altered with As^III^ treatment when compared with controls. Columns 2–4 show the fold change in metabolites compared with controls, and columns 5–8 show the means and standard deviations of the detected metabolites. IU and IU+ *n *= 8; CTRL and PN *n *= 7; *p ≤ 0.05, † *p* ≤ 0.1 compared with controls).							

**Figure 4:**
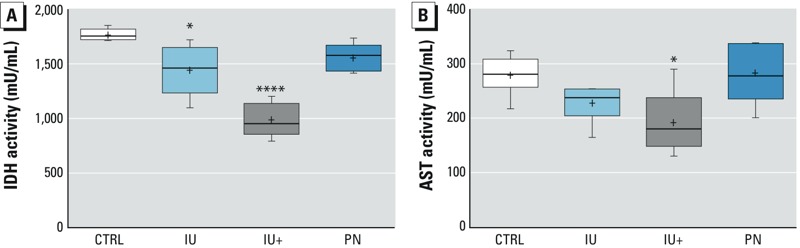
As^III^ exposure, TCA cycle, and AST enzymatic activity. (*A*) IDH activity was decreased in the livers of IU and IU+ treatment groups, but (*B*) AST activity was decreased only in the IU+ group. The box extends from the 25th to the 75th percentile, the whiskers show the entire distribution, the line is the median, and the + indicates the mean. (*A*) CTRL, IU+, and PN *n *= 5; IU *n *= 6; (*B*) CTRL, IU, and IU+ *n *= 6; PN *n *= 5; ****p ≤ 0.0001, *p ≤ 0.05 compared with controls.

*As^III^ and TCA cycle and transaminase enzymatic activity.* The activity of hepatic IDH was investigated to confirm the attenuation of TCA cycle activity observed in the plasma metabolomic analysis. IDH is upstream of isocitrate (the IDH substrate), then alpha-ketoglutarate, succinyl-CoA, succinate, fumarate, malate, and oxaloacetate. We observed decreased succinate, fumarate, and malate. IDH is reported to be inhibited by As^III^ (Higashi et al. 1965). A significant decrease in IDH activity was detected in livers from IU and IU+ mice (Figure 4A). A decrease in hepatic AST or ALT activity is associated with fatty liver disease (Pol et al. 1991). A significant decrease in hepatic AST activity was detected in the IU+ group (Figure 4B) with no change in the ALT activity. The fact that PN exposure to As^III^ did not significantly decrease IDH activity suggests that portions of the TCA cycle were inhibited in adult animals that were only exposed to As^III^ during fetal development. Therefore, *in utero* exposure to arsenic was a substantial contributor to disruption of the normal TCA cycle in adulthood.

**Figure 5:**
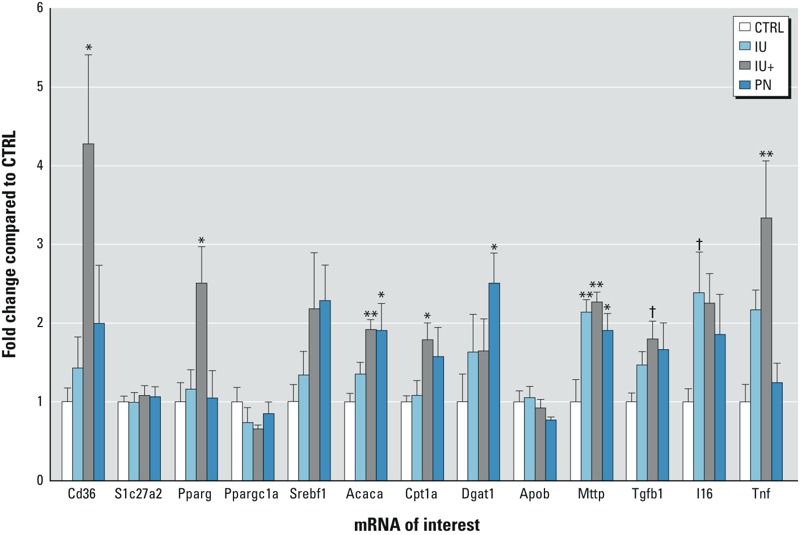
As^III^ exposure and the hepatic transcription profile. mRNA levels of lipid-handling and cytokine genes are shown as fold change compared with controls, and error bars represent the standard error. *n *= 5; *p ≤ 0.05, **p ≤ 0.01, ^†^*p* ≤ 0.1 compared with controls.

*As^III^ and the hepatic transcription profile.* The hepatic expression of several key genes involved in metabolism, lipid uptake, inflammation, and triglyceride export was investigated in order to determine the effects of As^III^ exposure on their expression and whether they contributed to disrupted lipid homeostasis (Figure 5). Expression of fatty acid uptake transporter *Cd36* was significantly increased only in the IU+ group. Furthermore, mRNA for *Pparg*, a master regulator of lipid and glucose metabolism, was increased in the IU+ group. Expression of *Acaca*, which encodes the enzyme that catalyzes the carboxylation of acetyl-CoA, was significantly increased in the IU+ and PN treatment groups. In addition, mRNA for *Cpt1a*, a key player in the β-oxidation of fatty acids, was significantly increased in the IU+ treatment group. The expression of *Dgat1*, an enzyme critical to the formation of triglycerides from acyl-CoA and diacylglycerol, was increased in livers from the PN mice only. Moreover, mRNA for *Mttp*, which plays an essential role in lipoprotein assembly, was significantly increased in the livers of all As^III^-treated mice. Expression of *Tgfb1*, a pro-fibrotic growth factor, showed a trend of increasing in the IU+ group, whereas mRNA for *Il6*, a cytokine implicated in NAFLD, showed a trend of increasing in the IU group. Finally, expression of *Tnf*, a cytokine that has been well established to play a role in the development of NAFLD, was increased in the IU+ group. These changes in gene expression in the livers of mice exposed to As^III^ *in utero* and in early life suggest disrupted lipid homeostasis and increased inflammatory mediators as a consequence of fetal exposure to low levels of arsenic.

## Discussion

**Figure 6:**
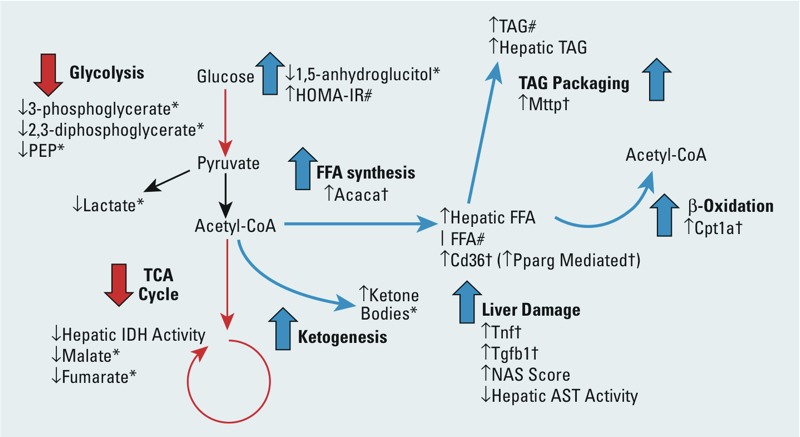
Schematic summary of the detected changes from developmental exposure to As^III^. Here, we present an abbreviated list of alterations observed in the IU+ group with a schematic representation of their relationships to each other. (↑ increased compared with controls; ↓ decreased compared with controls; | no change compared with controls; * data from week 9 blood plasma metabolomics; # data from week 5 blood plasma; † hepatic RT-qPCR data).

This study revealed the development of metabolic disease in mice after low-level As^III^ exposure during fetal development and postnatally. Figure 6 summarizes our findings with a simplified and condensed schematic of the relationships among metabolites, metabolic pathways, and genes, as well as general markers of liver damage with IU+ exposure. NAFLD is thought to be the hepatic manifestation of metabolic syndrome (Paschos and Paletas 2009). The detection of an increased incidence of NAFLD from developmental and continuous As^III^ exposure in conjunction with poor glycemic control and increased insulin resistance provides evidence for the induction of metabolic disease by arsenic (Table 2 and Figure 3B). However, it is also important to note that no increase in HbA1c was detected. When observed along with increased body weight and increased TAGs, these risk factors for cardiometabolic disease were restricted to the IU+ exposure group. Although it is possible that these changes were significant in the IU+ group because this group experienced the longest period of As^III^ exposure, other changes were also detected in the shorter-exposure (or pulsed-exposure) IU group but not in the postnatally exposed mice. These findings suggest that developmental exposure to arsenic is a primer for adult onset of disease, with the most severe pathology developing with continued presence of As^III^.

The concentration of As^III^ used in this study (100 ppb) is environmentally relevant in many areas of the United States where individuals and communities rely on nonmunicipal, often well-based, sources of water; in addition, areas in southeast Asia are exposed to As^III^ at parts per million concentrations from groundwater sources (Smedley and Kinniburgh 2002). However, the metabolism of arsenic was not examined in these mice, so there may be unaccounted-for differences in toxicokinetics. This study did include a female cohort; however, initial observations did not detect significant changes in total body weight or proportional liver weight. Therefore, males were analyzed to determine whether increases in liver and body weight were connected to metabolic pathology.

The links between As^III^ exposure and NAFLD have been established for adult exposures at higher As^III^ concentrations, but little is known about the mechanisms or the impact of low-level As^III^ on fetal and early developmental periods (Arteel et al. 2008; Reilly et al. 2014; Shi et al. 2014; Tan et al. 2011). Our laboratory has previously demonstrated the incidence of NAFLD in mice with *in utero* exposures to 100 ppb As^III^ (Sanchez-Soria et al. 2014) and, to our knowledge, is the only known example of this phenomenon. The present work has determined a mechanistic basis for the effects of arsenic on metabolic dysregulation and disease.

Starting at 5 weeks of age, the IU+ group was overweight when compared with controls and remained so for the duration of the study (Figure 3A). Differential changes in weight with As^III^ exposure have been demonstrated, including decreased birth weights for mice exposed to low levels of As^III^ *in utero* (Kozul-Horvath et al. 2012; Sanchez-Soria et al. 2014). However, it has recently been reported that obese adolescents appeared to metabolize arsenicals less efficiently than their normal-weight counterparts, suggesting a link between obesity and susceptibility to arsenical toxicity (Su et al. 2012). Continuous As^III^ exposure increased plasma TAG concentrations (Figure 3C), a finding that is contrary to those of earlier studies examining *in utero* exposure to arsenic. Sanchez-Soria et al. (2014) found a decrease in plasma TAG at 4 months in Swiss Webster mice on a regular diet that were exposed to 100 ppb As^III^ *in utero*. Similarly, Srivastava et al. (2007) found decreased plasma TAGs at 10 weeks in ApoE ^–/–^ mice on a standard diet that had been exposed to 49 ppm As^III^. Although the exact mechanism is unclear, continuous exposure to As^III^ while consuming a Western-style diet has an additive effect to increase plasma TAG, which may explain the differences between previous studies and the present one.

The extent of NAFLD resulting from developmental exposure to low levels of As^III^ was substantial and appeared to be greatest in the As^III^ treatment groups with *in utero* and continuous exposure (Figure 2). The increased proportional weight, TAGs, FFAs, and cholesterol detected in the IU (TAGs and proportional weight only) and IU+ groups are strongly indicative of an NAFLD state induced by developmental exposure to As^III^ that is most severe with continued exposure (Figure 1A–D) (Puri et al. 2007; Simonen et al. 2011). Surprisingly, we detected no increase in FFAs in blood plasma; such an increase is typically associated with fatty liver disease. We suspect that this may be due to the observed NAFLD being the result of altered energy metabolism in the liver rather than to an overall increase in circulating FFAs (Zhang et al. 2014). We intended to examine ALT and AST activity in plasma, but the small size of the plasma samples limited our ability to probe all biochemical targets. Hepatic transaminase activity was examined as a surrogate indicator of liver damage, and decreased AST activity was detected in the IU+ group (Figure 4B) with no change in ALT activity (data not included). Elevated AST and ALT activity in plasma are correlated with liver injury, whereas decreased hepatic AST and ALT activity have been reported in fatty liver disease (Pol et al. 1991). Therefore, the observed decrease in AST activity suggests more severe NAFLD in the IU+ group.

The metabolomic data suggest disruption of the TCA cycle because decreased levels of TCA intermediates were detected in the groups exposed to As^III^ *in utero* (Table 2). Pyruvate dehydrogenase, α-ketoglutarate dehydrogenase (Bergquist et al. 2009), IDH (Higashi et al. 1965), and succinate dehydrogenase (Hosseini et al. 2013) are known to be disrupted by arsenicals. In this regard, hepatic IDH activity was significantly reduced in the IU and IU+ animals (Figure 4A), supporting disruption of the TCA cycle by developmental As^III^ exposure. Because of sample size limitations, other hepatic dehydrogenases involved in the TCA cycle were not analyzed, but future studies should determine whether these enzymes are targets of developmental As^III^ exposure. Persistent disruption of the TCA cycle has severe repercussions on homeostasis in humans (Rustin et al. 1997). To our knowledge, the continued inhibition of these dehydrogenases after *in utero* As^III^ exposure is a novel finding. It is unlikely that this continued decrease in activity is a result of direct As^III^ inhibition but is instead a disruption of developmental programming. This hypothesis is supported by the lack of IDH inhibition in the PN group and by the remaining activity decrease in the IU group long after As^III^ was removed. This observation reinforces the argument that developmental exposure is a primer for adult disease, particularly in the context of continuous exposure after birth.

The role played by arsenic in the disruption of glycolysis, that is, replacing phosphate in the reaction catalyzed by glyceraldehyde 3-phosphate dehydrogenase (GAPDH), has been well established (Hughes 2002). The changes detected in glycolysis may be a result of GAPDH disruption; however, the concentrations of As^III^ that have been shown to disrupt GAPDH are much higher than 100 ppb. It has also been shown recently that As^III^ induces the Warburg effect in multiple human cell lines. Specifically, there is an increased production of lactate and an increase in the expression of glycolysis-related genes with low-level As^III^ exposure (Zhao et al. 2013). Given these findings, and given that arsenicals are also potent inhibitors of pyruvate dehydrogenase, an increase in lactate was expected to occur in As^III^-treated mice (Schiller et al. 1977). However, significantly reduced lactate levels were detected in the IU and IU+ groups as well as an increase in pyruvate in the PN group (Table 2). Thus, there was a significant difference between *in utero* (continuous or noncontinuous) and postweaning exposure. Increased pyruvate levels could suggest decreased pyruvate dehydrogenase activity (due to known As^III^ inhibition or to increased acetyl-CoA negative feedback) or decreased lactate dehydrogenase activity, which is unlikely, owing to a strong association between lactate dehydrogenase activity and arsenical exposure (Liao et al. 2012). An increase in LDH activity caused by arsenic would also suggest that the decrease in lactate observed in the IU and IU+ groups was unlikely to have been a result of decreased lactate dehydrogenase activity, so the mechanism behind this decrease is not readily apparent. The inhibition of pyruvate dehydrogenase by negative feedback inhibition with acetyl-CoA is consistent with our proposed mechanism of TCA cycle disruption; however, PN exposure did not appear to substantially disrupt the TCA cycle. The mechanism behind the observed increase in pyruvate in the PN group but not in the *in utero*–exposed groups is unclear. Ongoing exposure to As^III^ in the PN group could have decreased pyruvate dehydrogenase activity, but a similar effect would be expected to occur in the IU+ group, which was not the case. With developmental exposures, it is unlikely that the alterations in enzymatic activity are a result of direct disruption by As^III^, but rather are some form of altered developmental programming. Follow-up studies examining the effects of developmental exposure on energy metabolism are recommended to better resolve the complex and diverse changes that appear to be occurring.

The observed decrease of select LCPUFAs in IU plasma only (Table 2) is puzzling because it represents one of the few changes present in only the IU treatment group. LCPUFAs, as polyunsaturated fatty acids, are capable of activating a variety of peroxisome proliferator-activated receptors (Ppars) (Grygiel-Górniak 2014), resulting in decreased activity of Srebf1 (Yoshikawa et al. 2002). Decreased LCPUFAs in the liver have been shown in patients with NAFLD, and it has been suggested that this decrease may be partially responsible for the development of NAFLD through decreasing suppression of SREBF1 activity and attenuating activation of peroxisome proliferator-activated receptor alpha (PPARA). These changes result in conditions where TAG and FFA synthesis are favored over FFA oxidation and TAG export (Araya et al. 2004). Although we did not examine *Ppara*, we did detect expression of *Srefbf1* and *Pparg*, and we observed an increase in the expression of *Pparg* in the IU+ group, but we saw no statistically significant change in *Srebf1* (Figure 5). Patients with NAFLD have increases in the expression of both of these genes, and it is thought that they work together to potentiate the lipogenic state in NAFLD (Pettinelli and Videla 2011). As^III^ is also capable of decreasing *Pparg* expression and signaling, resulting in disruption of adipogenesis (Wauson et al. 2002) and thereby contributing to the observed pathology; however, additional investigation will be needed to validate this mechanism.

We propose that As^III^-mediated disruption of the hepatic TCA cycle leads to a loss of normal acetyl-CoA flux that results in a push towards ketogenesis, as shown in the plasma metabolomic findings and in the increased expression of FFA synthesis– and β-oxidation–related genes (Table 2 and Figure 5). The elevated expression of *Acaca* in the IU+ and PN groups suggests increased FFA synthesis, and the increase in expression of *Cpt1a* in the IU+ group suggests an increase in β-oxidation (Figure 5). However, it is important to note that the product of *Acaca*, malonyl-CoA, is an inhibitor of *Cpt1a*, which prevents β-oxidation and FFA synthesis from occurring simultaneously (Paulson et al. 1984). We propose that expression of these enzymes is increased in order to handle increased acetyl-CoA, in the case of *Acaca*, and that expression of *Cpt1a* is increased in order to reduce FFA-induced damage in a hepatoprotective manner. It is likely that increased FFA synthesis and β-oxidation are occurring, but their temporal activity differs. Increased expression of *Acaca* in the groups with ongoing exposure suggests that this change may be dependent on the continued presence of As^III^. Increased expression of *Mttp* was also detected in all As^III^-treated groups, but *Dgat1* was increased only in the PN group (Figure 5). This finding suggests increased TAG export in all As^III^-treated groups but only increased TAG synthesis in the PN group, which may have been caused by unexamined changes downstream of transcription. We also observed increased expression of *Cd36* in the IU+ treatment group, which traditionally would suggest an increase in FFA uptake, but this finding is inconsistent with the observed plasma FFA levels, which remained unchanged. However, it has recently been shown that *Cd36* is important in the regulation of very low density lipoprotein (VLDL) secretion in the liver, which may suggest a protective role against hepatic steatosis (Nassir et al. 2013). In addition, expression of cytokines involved in NAFLD and fibrosis were examined, and in the IU+ group, there was a trend towards an increase in *Tgfb1* (Hasegawa et al. 2001) and an increase in *Tnf* (Crespo et al. 2001), both of which are indicative of more severe NAFLD, oftentimes pointing to NASH. Expression of *Il6* was also examined, and a trend towards an increase was observed only in the IU group; this finding cannot be explained considering that the most severe pathology appeared in the IU+ group, and *Il6* is associated with greater inflammation and fibrosis in NAFLD (Wieckowska et al. 2008). Taken together, many of these gene expression changes are consistent with NAFLD and suggest increased hepatic acetyl-CoA.

## Conclusions

In summary, *in utero* and postnatal exposure to low levels of As^III^ combined with a Western-style diet promoted NAFLD and increased risk factors for cardiometabolic disease in male Swiss Webster mice. As^III^ exposure during fetal development mediated disruptions in energy metabolism. Evidence for metabolic dysfunction includes altered metabolites of glycolysis as well as a disruption of the TCA cycle due in part to decreased IDH enzymatic activity. We believe that acetyl-CoA is likely overwhelming the liver owing to an impaired TCA cycle, resulting in a push towards ketogenesis and an increase in expression of FFA synthesis, β-oxidation, and TAG synthesis and export genes.

IU+ exposure resulted in the most severe pathology, and this group was the only one to see a substantial increase in risk factors for cardiometabolic disease, including obesity, hyperglycemia, insulin resistance, and increased plasma TAGs. However, based on other metabolomic changes that were observed, the severity of the increase was not necessarily a result of longer exposure because distinct changes in metabolic endpoints were observed when comparing the *in utero* and PN cohorts. Many changes were only detected in the IU or IU+ exposure groups and not in the the PN group, suggesting that fetal developmental exposure to As^III^ provides unique alterations to energy metabolism that result in susceptibility to disease later in life that is most severe with continuous exposure to As^III^ after birth.

## Supplemental Material

(100 KB) PDF
