# Supplementary material for: Effects of Arsenite Exposure during Fetal Development on Energy Metabolism and Susceptibility to Diet-Induced Fatty Liver Disease in Male Mice
Source: Environ Health Perspect. 2015 Jul 7;124(2):201–9. doi: 10.1289/ehp.1409501 (PMC4749082; doi:10.1289/ehp.1409501)
Supplement: (100 KB) PDF [file ehp.1409501.s001.acco.pdf]

Environ Health Perspect
DOI: 10.1289/ehp.1409501

Note to Readers: *EHP* strives to ensure that all journal content is accessible to all readers. However, some figures and Supplemental Material published in *EHP* articles may not conform to 508 standards due to the complexity of the information being presented. If you need assistance accessing journal content, please contact ehp508@niehs.nih.gov. Our staff will work with you to assess and meet your accessibility needs within 3 working days.

Supplemental Material

Effects of Arsenite Exposure during Fetal Development on Energy Metabolism and Susceptibility to Diet-Induced Fatty Liver Disease in Male Mice

Eric J. Ditzel, Thu Nguyen, Patricia Parker, and Todd D. Camenisch

Table of Contents

Table S1. Litter Information

Table S2. Male Mice Litter Contribution to Exposure Groups

Table S1. Litter Information

Litter	Treatment	Males	Females	Total
1	PN	4	5	9
2	CTL/PN	6	5	11
3	CTL	8	3	11
4	IU	4	6	10
5	IU	8	3	11
6	IU	4	6	10
7	IU+	4	0	4
8	IU+	7	1	8
9	IU+	5	5	10

Nine litters were utilized in this study, 1-3 were not treated with As (III) during development, but 4-9 were. After birth litters 7-9 continued to receive As (III) through drinking water. After weaning, mice assigned to the PN group began to receive As (III) through the drinking water.

Table S2. Male Mice Litter Contribution to Exposure Groups

Litter	CTRL	IU	IU+	PN
1	0	0	0	3
2	2	0	0	2
3	8	0	0	0
4	0	4	0	0
5	0	7	0	0
6	0	3	0	0
7	0	0	3	0
8	0	0	6	0
9	0	0	4	0

The number of male mice included in each exposure group from each litter is listed above.
